# Immunomodulation of Homeopathic Thymulin 5CH in a BCG-Induced Granuloma Model

**DOI:** 10.1155/2013/686018

**Published:** 2013-01-28

**Authors:** Leoni Villano Bonamin, Cesar Sato, Ruggero Zalla Neto, Graziela Morante, Thayná Neves Cardoso, Fabiana Rodrigues de Santana, Cideli de Paula Coelho, Lika Osugui, Ana Flavia Popi, Elizabeth Cristina Perez Hurtado, Mario Mariano

**Affiliations:** ^1^Research Center of Universidade Paulista, Rua Dr. Bacelar 1212, 5th Floor, 04026-002 São Paulo, SP, Brazil; ^2^Laboratory of Veterinary Pathology, Universidade de Santo Amaro, São Paulo, SP, Brazil; ^3^Instituto de Ensino, Pesquisa e Desenvolvimento Royal, São Roque, SP, Brazil; ^4^Laboratory of Immunology, Universidade Federal de São Paulo, São Paulo, SP, Brazil

## Abstract

The present study analyzed the immune modulation mechanisms of thymulin 5CH in a granuloma experimental model. Male adult Balb/c mice were inoculated with BCG into the footpad to induce granuloma, which was quantitatively evaluated. The phenotypic characterization of phagocyte, T- and B-lymphocyte populations in the peritoneum, and local lymph node was done by flow cytometry. During all experimental periods, thymulin 5CH and vehicle (control) were given *ad libitum* to mice, diluted into the drinking water (1.6 × 10^−17^ M). After 7 days from inoculation, thymulin-treated mice presented reduction in the number of epithelioid cytokeratine-positive cells (*P* = 0.0001) in the lesion, in relation to young phagocytes. After 21 days, the differentiation of B1 peritoneal stem cells into phagocytes reached the peak, being higher in thymulin-treated mice (*P* = 0.0001). Simultaneously, the score of infected phagocytes in the lesion decreased (*P* = 0.001), and the number of B1-derived phagocytes, CD4+ and CD8+ T lymphocytes in the local lymph node increased in relation to control (*P* = 0.0001). No difference was seen on the CD25+ Treg cells. The results show that thymulin 5CH treatment is able to improve the granuloma inflammatory process and the infection remission, by modulating local and systemic phagocyte differentiation.

## 1. Introduction

The experimental demonstration of the effects of high diluted homeopathic medicines has been the focus of several studies using animal models in the last ten years, providing enough substrate to recent systematic reviews [1–4], including those about immunology and homeopathy [5–10]. Among these studies, a wide range of effects related to the administration of endogenous substances prepared as homeopathic medicines has been observed by our group in different animal species [11–14].

Thymulin is an endogenous peptide with immune regulatory function that has been studied as homeopathic high dilutions since the eighties [15–18]. In these studies, several evidences were highlighted about the modulation property of homeopathic thymulin prepared at 5CH potency, including seasonal variations and variable effects according to individual previous status [17, 18]. In C57BL/6, high diluted thymulin (10^−11^ mg/animal) reduced the cell immune response. Nonetheless, in NZB mice, that develop lupus spontaneously, the same thymulin concentration decreased the humoral thymus-dependent response, decreasing self-immunity potential of lymphocytes harvested from these animals [16].

Recent studies made by our group showed that thymulin 5CH offered *ad libitum* into the drinking water to mice bearing Ehrlich tumor, decreased partially the suppression of the bone marrow after cyclophosphamide treatment, and increased bone metaplasia area into the tumor [19]. In broiler chicken, thymulin 5CH offered into the drinking water since the first day of life improved the weight gain, survival, and productivity indexes at slaughter time [13]. Important to point out that offering homeopathic medicines into the drinking water represents a good, cheap, and easy tool to treat farm animals [20].

Thymulin is produced by the thymus epithelial component and is composed by nine aminoacids (Glp-Ala-Lys-Ser-Gln-Gly-Gly-Ser-Asn-OH). It is important in several steps of T-lymphocyte maturation [21, 22] and in several neuro-endocrine functions, including sexual maturity, reproduction [23, 24], and neuroprotective functions [25, 26]. Zinc is an important cofactor in thymulin physiological activity, being controlled by metallothioneins [24, 27]. Several pathological processes can be influenced by physiological concentrations of thymulin, such as T cell and phagocyte function recovery in immune suppressed animals [28, 29], balance of cytokines [29–31], and improvement of regenerative functions [32]. However, all these effects refer to physiological quantities of serum thymic factor that are quite higher than those found in homeopathic preparations and their effects cannot be directly associated. This fact points toward to an important question: are the immune modulation effects of homeopathic serum thymic factor demonstrable?

The aim of the present study is to demonstrate the modulation properties of homeopathic 5CH thymulin using the experimental BCG-induced granuloma model. This model was chosen due to its well-established process to study immune-mediated chronic inflammation and the role of the pertinent cells, including phagocytes, T, B1, and B2 cells [33–38].

## 2. Materials and Methods

### 2.1. Animals

Specific pathogen free (SPF) adult male BALB/c mice (25 to 35 g of body weight) were obtained from a reference rodent biotery CEDEME/UNIFESP, São Paulo, and maintained standing in the Research Center SPF biotery of Paulista University during at least seven days before any experimental procedure, due to environmental adaptation. During all experimental period, animals were kept in conventional microisolators (Techniplast), being five animals per cage. Sterile food and water were offered *ad libitum*. The controlled temperature was 22 ± 2°C and light was programmed to be 12 hours light, from 06:30 h to 18:30 h. The number of air cycles per hour inside cages was 75/hour, being the humidity between 50 and 65%.

### 2.2. Thymulin Preparation

The thymulin 5CH was prepared from the stock commercial homeopathic preparation Thymulin 4CH obtained from Boiron Brasil Laboratories, which was prepared from the zinc free synthetic peptide or serum thymic factor (MW = 858.85), whose purity degree was 98.66% (according to the supplier). The dilution and stocking techniques were done according to the French Pharmacopeia for homeopathic products, which means that the first 1 : 100 dilution (w/v) of pure serum thymic factor was used to produce the 1CH potency after 100 succussions; thus, 1 : 100 dilution (v/v) plus 100 succussions of 1CH was used to produce 2CH. The 3 and 4CH potencies were prepared similarly. The vehicle used was 70% hydroalcoholic solution and the final product was stocked at room temperature.

The disposable thymulin 5CH potency solution was prepared just before use, diluting 1 : 100 the Thymulin 4CH matrix into 15 mL of 30% hydroalcoholic solution, using conventional amber flasks and an automatic mechanical system to perform 100 succussions (Autic). A parallel similar preparation was done using only succussed 30% hydro-alcoholic solution, to be offered to the control (vehicle) mice. 

All technical procedures were in accordance to the Brazilian Homeopathic Pharmacopeia, 2nd Edition and to the French Pharmacopeia-Homeopathy, 6th Edition.

To be sure that there was no zinc contamination of medicines, both thymulin 5CH and vehicle (succussed 30% hydroalcoholic solution) samples were sent to a certified laboratory (CBO Analysis, Brazil) in order to measure its quantity. No significant amount of zinc was found in both vials.

### 2.3. Blinding

After the drug preparations, another technician who did not participate of the experimental procedures changed the labels of each flask to another written A or B, and the same codes were written in a sheet of paper together with the original labels. This sheet was closed in a sealed envelope and kept up to the end of statistical analysis, when codes were broken.

### 2.4. Treatment

Mice received A or B treatment in a free access into the drinking water. Thus, 0.1 mL of A or B preparation (thymulin 5CH or vehicle 5CH) was mixed regularly to 250 mL of drinking water into an appropriate sterile bottle, in order that the final theoretical thymulin concentration offered to mice was 4 × 10^−13^ M. This concentration was chosen based on that previously tested by us, using broiler chickens [13]. The water and the added medicine were replaced daily, during all experimental period, from the day of BCG inoculation to the day of mice necropsy.

Each group (A or B) was composed by 10 to 15 mice, according to the experimental time and procedure.

### 2.5. BCG Inoculation and Observation of Lesions

The Calmette-Guérin bacilli (Vacina-BCG, Brazil) were supplied by Butantan Institute, São Paulo, containing 2 × 10^6^ viable bacteria (CFU) by milligram of a lyophilized powder. The powder was diluted into apyrogenic sterile saline to get 2 × 10^5^ CFU in 50 *μ*L at the moment of inoculation. The inoculation was made in the subcutaneous tissue of the left footpad of mice, using an ultra-fine shot (BD Medical, USA).

Before inoculation, at zero time, the paw thickness was measured by a digital micrometer (Mitutoyo IP65, Japan). Then, regular measurements were made in the subsequent days to analyze the macroscopic inflammation evolution. The granuloma growth and the respective local and systemic immunological parameters were studied after 7, 14, and 21 days after BCG inoculation, when batches of animals were euthanized in a CO_2_ chamber. The paw, spleen, popliteal local lymph node, and peritoneal fluid were harvested and manipulated according to specific experimental procedures.

### 2.6. Histopathology and Histomorphometry

The subcutaneous tissue of the paw, the spleen, and the local popliteal lymph node were fixed in 10% buffered formalin for no more than 24 hours to avoid antigen masking. The histological procedures were made according to the conventional hematoxylin-eosin (HE), Ziehl-Neelsen (ZN), Pearls (P), and immunohistochemical staining procedures.

**Figure 1:**
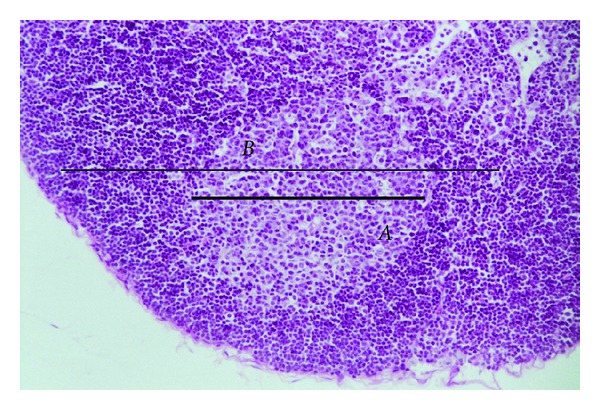
Photomicrograph of a popliteal lymph node from a thymulin 5CH treated mouse, after 21 days of BCG inoculation. The major axis (*B*) represents the follicle diameter and the minor axis (*A*) represents the germinal center. HE staining, magnification 1 : 200.

From HE stained slides, a qualitative-descriptive analysis of the granuloma structure was made for the footpad and a quantitative analysis of the follicle diameter (*B*) and the respective germinal center (*A*) of the local lymph node was made using histometry technique (Figure 1). In this case the major axis of each structure was measured using a 20 times objective lens and a special 10x ocular lens, graduated by a 100 points scale, being 100 equal to 1 mm. The final percentage values were obtained by the following formula:

(1) $$\begin{array}{c} P=\frac{A\times 100}{B}. \end{array}$$

Since the descriptive analysis of spleen stained by HE method revealed a trend in treated animals to present bigger follicle area and an important quantity of golden pigment, suggestive of hemosiderin, two specific histometric analyses were made in this case: (a) an additional Pearls staining method specific for iron pigments was used in the same material to analyze the number of positive macrophages per field in the spleen parenchyma, using a 40x objective. Ten random fields were analyzed per slide; (b) the mean area of spleen follicles per slide was calculated using photomicrographs of HE stained slides at magnification 1 : 100 and the Image J software for Windows.

**Figure 2:**
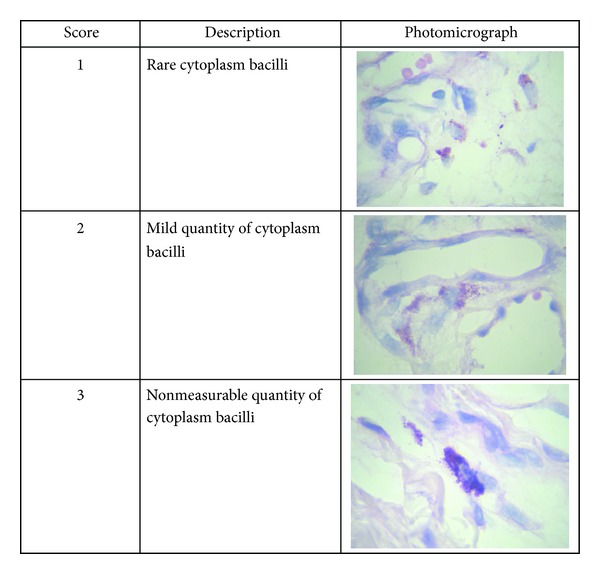
The score diagram of BCG phagocytosis in macrophages located in the BCG inoculation site. ZN staining, magnification 1 : 1000.

From the slides stained by ZN method, the histometry was made by scores representing the quantity of bacilli into the phagocyte cytoplasm. Twenty macrophages by field were randomly analyzed using a 1000x immersion objective and scores were attributed to each cell by two independent observers to avoid subjective bias in the analysis (Figure 2).

### 2.7. Immunohistochemistry and Histomorphometry

All samples of subcutaneous tissue presenting granuloma were prepared with a 5 microns paraffin embedded tissue slices and paraffin removal was made through two baths in absolute xylol and two baths in absolute alcohol, three minutes each. Slides were, then, washed in current water.

The antigen retrieving was made using heat treatment into citrate buffer (DAKO), using an electric pot at 80°C (PANASONIC) for twenty minutes. Then, slides were washed in PBS, pH = 7.2 (SIGMA), during five minutes in agitation, dried, and the tissue sample was delimited with a Pap-pen (AbCAM). The endogenous peroxidase activity was blocked by slides incubation in 5% H_2_O_2_ in methanol, during fifteen minutes at room temperature. After a new washing in PBS, slides were treated with 2.5% of normal horse serum (VECTOR) during twenty minutes at room temperature for blocking of unspecific antigen adsorption sites and tissues were immediately treated with the primary antibody, whose dilution are described in Table 1. This incubation was made at 4°C, overnight, in a humid chamber. All dilutions of primary antibody were made using specific diluent for immunohistochemistry (DAKO).

In the day after, cuts were washed in PBS (SIGMA) for six minutes and treated with the polymer-peroxidase conjugated secondary antibody (VECTOR), for rat or rabbit IgG, according to the origin of the primary antibody. This incubation lasted 30 minutes, at room temperature. After new washing in PBS, they were exposed to DAB (DAKO) for few seconds until the appearance of the brown color, washed in tap water, counter-stained with Harris hematoxylin, and mounted. The negative control of reactions was made by changing the primary antibody by the diluent alone before incubation. The positive control was made using mouse lymphoid tissue samples to test leukocytes markers and the epidermis itself, for testing the anticytokeratin antibody reactivity.

### 2.8. Histomorphometry

The histomorphometry was performed to better identify and quantify immune cells (Table 1) in the lesion, since the HE observation was only descriptive and the flow cytometry of paw subcutaneous tissue cell suspension was not possible to be done, due to the impossibility to define cell populations with a minimal number to make them recognizable, even if a pool of several footpads cell suspension was done.

The evaluation of the chronic inflammation in the inoculated subcutaneous tissue by the incidence of specific leukocyte subsets was made by counting the number of positive cells per field for each marker (Table 1), in which ten random fields were chosen in the connective tissue of the footpad and analyzed at the magnification of 1 : 1000. Tissues were harvested from mice inoculated after 21 days, since the main changes in lesion progression and cell migration observed in the flow cytometry were related to this time, exception to the cytokeratine positive epithelioid cells, which presented the more expressive changes after 7 days of inoculation.

### 2.9. Flow Cytometry

Two kinds of samples were submitted to flow cytometry: in a first set of experiments, peritoneal washing fluids were obtained, for B1/B2 cells counting; in a second set of experiments, the local lymph node single cell suspension was obtained, for B1/B2 and T-cell subsets counting.

In the first set, mice were euthanized using a CO_2_ camera in the 7, 14, and 21 days after BCG inoculation and the peritoneal washing was made just after this procedure. Thus, 5 mL of RPMI medium (GIBCO) were injected into the peritoneal cavity and a smooth massage was made to allow the release and homogenization of cells. The washing fluid was harvested using a plastic Pasteur pipette, transferred to plastic 15 mL Falcon tubes immersed in ice, and centrifuged at 2000 rpm for 5 minutes.

Since the relevant results obtained from the first set of experiments revealed that the cell changes occurred only in 21 days after BCG inoculation, in the second set, regarding the lymph node cell counting, mice were euthanized just after this time. Thus, in the second set of experiments, mice were euthanized and the popliteal lymph node was harvested and put inside a drop of 2% PBS/BFS on a Petri plate, maintained in an ice tray. The cells were suspended with the help of a scalpel and the suspension was washed with 2% PBS/BFS and transferred into a 15 mL Falcon plastic tube kept immersed in ice. To obtain enough cells to be measured, five lymph nodes from the same group were used to complete a single tube and the procedure was repeated twice, in two repetition blocks. For each tube, the volume of samples was adjusted to 1.5 mL and 0.5 mL of liberase (ROCHE) previously diluted at 0.1 mg/mL was included. The tubes were maintained under agitation at 37°C for 30 minutes, in order to get a complete cell release from tissue debris. After this procedure, the samples were homogenized and filtered to another tube, using a 70 micra cell filter (BD) and, then, centrifuged.

After centrifugation, cellular pellets from peritoneal fluids and regional popliteal lymph node were suspended in 1 mL of ACK hemolytic buffer (GIBCO) and incubated for 2 minutes at room temperature. Then, 5 mL of PBS were added and the number of viable cells was counted in a modified Neubauer chamber (HAUSSER SCIENTIFIC).

 Cells (1 × 10^6^ cells per tube) were centrifuged and incubated with anti-mouse CD16/CD32 diluted 1 : 100 in PBS with 1% bovine serum albumin (PBS/BSA) for 20 minutes at 4°C, to perform the Fc*γ* II and III blocking. Then, peritoneal fluids and regional popliteal lymph node samples were washed and suspended in 200 or 300 *μ*L of PBS/BSA, being equally divided in two or three microtubes, respectively. The first microtube was incubated without antibodies and the second contained 1% anti-CD19 APC, anti-CD23 FITC, anti-CD5 PerCP, and anti-CD11b PE. The third microtube contained 1% anti-CD25 AF488, anti-CD4 PE, anti-CD19 PE Cy5.5, and anti-CD8 AF 405 (all antibodies from INVITROGEN). All of them were incubated for about 30 minutes at 4°C. The last centrifugation was done and the cells were suspended in 1 mL of PBS, fixed in 1% paraformaldehide (MERCK), and analyzed by flow cytometry using FACSCalibur (BD Biosciences, Mountain View, CA, USA) or the ATTUNE Acoustic Focusing Cytometer (APPLIED BIOSYSTEM). The Flow Jo 8.7 and the Attune 1.2 software were used to analyze data, respectively. Ten thousand events were acquired for each sample. The transposition of data to EXCEL 2007 and INSTAT 3.0 software was made after all analysis was completed and the raw data were checked by a third person.

**Figure 3:**
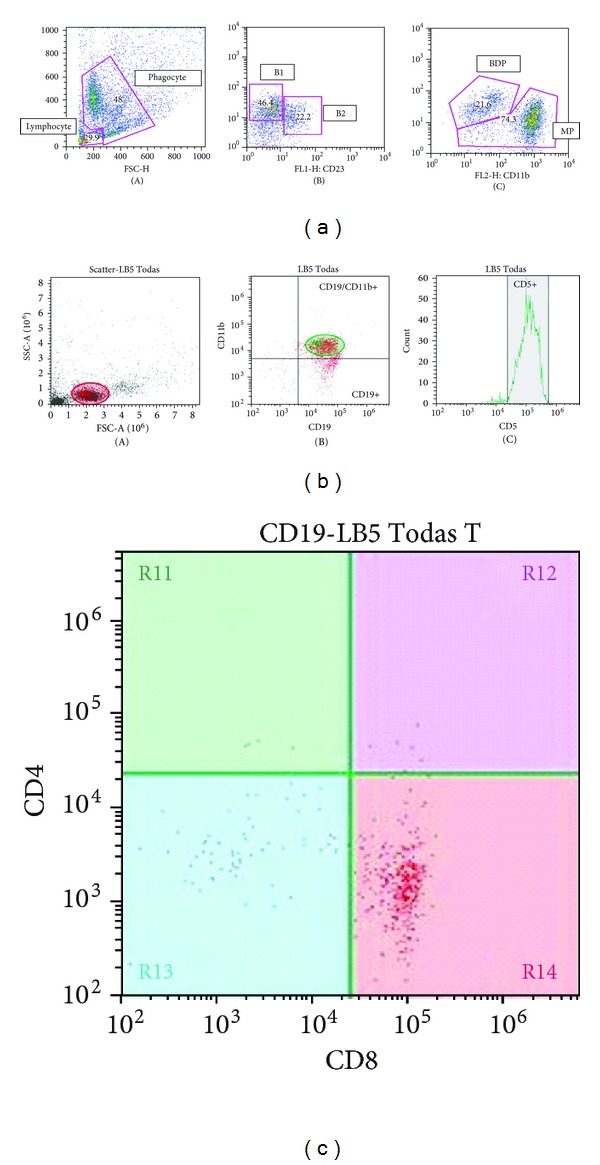
(a) Illustrative scattering diagram representing the percentage, distribution, and selection of B-cells populations in Flow cytometry of peritoneal washing fluid harvested from a control mouse. (A) lymphocyte and phagocyte gates identified on the basis of cell size (*y*-axis) and lumpiness (*x*-axis); (B) from the lymphocyte gate, a secondary diagram was extracted representing B1 (CD19+ CD23−) and B2 (CD19+ CD23+) cells; (C) from the phagocyte gate, another secondary diagram was extracted, representing B1 derived phagocytes—BDP (CD11b+ CD19+) and mature phagocytes—MP (CD11b+ CD19−). Graphs obtained from Flow Jo 8.7 software. (b) Illustrative scattering diagram representing the distribution and selection of B-cells populations in Flow cytometry of local lymph node cell suspension from a thymulin 5CH treated mouse. (A) lymphocyte gate identified after the cell size (*y*-axis) and lumpiness (*x*-axis); (B) from the lymphocyte gate, a secondary diagram was extracted representing B1 (CD19+ Cd11b+) cells; (C) from the B1 gate, another secondary diagram was extracted, representing B1a (CD11b+ CD5+) cells. Graphs obtained from Attune 1.2 software. (c) Illustrative scattering diagram representing the distribution and selection of T-cell subpopulations in Flow cytometry of lymph node cell suspension, from a thymulin 5CH treated mouse. The diagram was extracted from the lymphocyte gate. The predominant population was CD8+ cells (R14). Graphs obtained from Attune 1.2 software.

For both, peritoneal and lymph node cells, lumpiness and cell size were selected to determinate the lymphocyte and phagocyte subpopulations. The fluorescence obtained by the markers was used to establish the specific gates of positive cells. Thus, from the lymphocyte gate, the B2 cells were determined by the simultaneous high expression of CD19 and/or CD23 and the B1 cells were determined by the high expression of CD19 and CD11b. From this last subpopulation, the quantity of CD5 positive (B1 a) and CD5 negative (B1 b) cells was calculated. From the phagocyte gate, double positive cells for CD19 and CD11b were labeled as “B1-derived phagocytes” (BDP) and CD11b positive—CD19 negative cells were labeled simply as “mature phagocyte” (Figure 3(a)).

The analysis of T-cells subsets present in the lymph nodes was made from the lymphocyte gate. The CD19 negative cells were delimited in a specific gate from which the CD4 and CD8 cell subpopulations were determined. An additional counting was made from each of these subpopulations to determine the percentage of T reg CD25 positive cells (Figures 3(b) and 3(c)).

### 2.10. Statistical Analysis

The data were first analyzed by the Bartlett test to verify the homogeneity of samples and, then, submitted to Student *t*-test or Mann-Whitney test according to the case. The *X* ^2^ test was used to evaluate the proportion between cell subsets observed in the flow cytometry and histomorphometry. The granuloma evolution and histomorphometric parameters recorded in function of time were evaluated by MANOVA. In all cases, the values of *P* ≤ 0.05 were considered significant in relation to control.

## 3. Results

### 3.1. Gross Measurement of Granuloma Lesion

**Figure 4:**
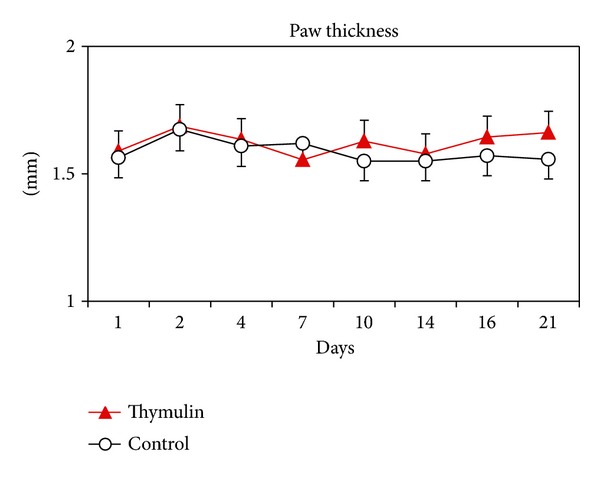
Evaluation of gross pathology (mean/standard deviation) in function of time of the granuloma size in the footpad of mice treated or not with thymulin 5CH. The paw thickness was measured with a micrometer. *P = 0.01, MANOVA.

The granuloma was macroscopically measured in function of time. The gross lesion in thymulin 5CH treated mice was bigger than control from the 10 day after inoculation, being the peak at 21 days (Figure 4).

### 3.2. Histopathology

**Figure 5:**
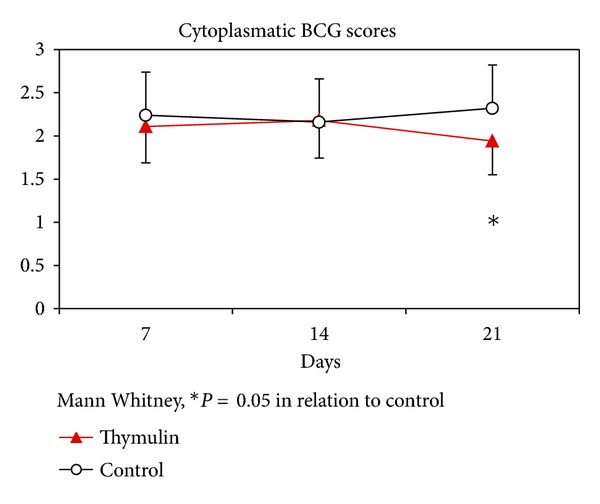
Evaluation of BCG infection scores in function of time obtained from Ziehl-Neelsen stained slides. Twenty phagocytes per field were analyzed by two independent observers, in blind. *P = 0.05, Mann-Whitney, and in relation to control. Data represent mean and standard deviation.

**Figure 6:**
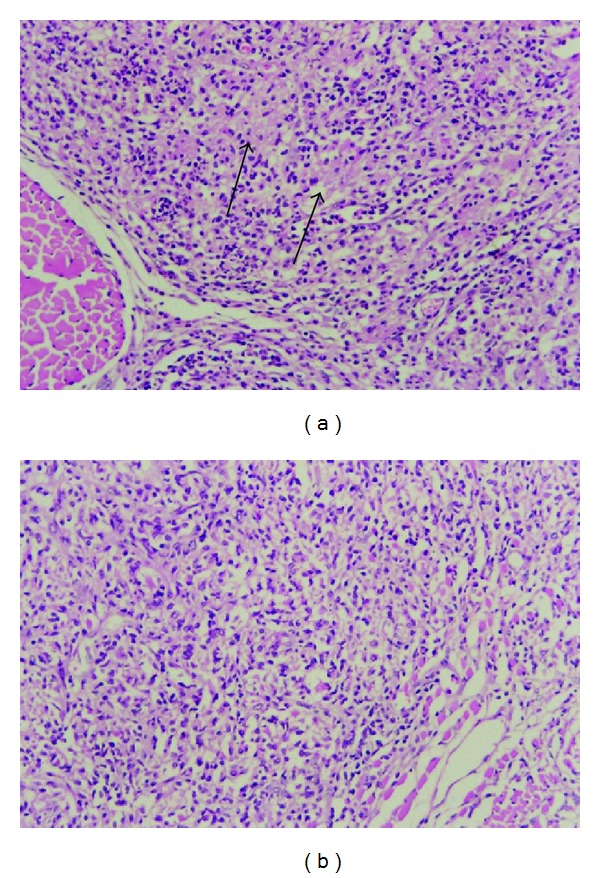
Photomicrography of granuloma at the 7 day after BCG inoculation into subcutaneous tissue. Note the presence of epithelioid cells (arrows) predominantly in the control group (a) and young phagocytes in thymulin 5CH treated group (b). Hematoxylin-eosin, magnitude 1 : 100.

The scores of cytoplasmatic BCG content obtained from Ziehl-Neelsen staining method revealed that, at the 21 day after inoculation, the thymulin 5CH treated mice showed significant reduction of infection in relation to control (Figure 5), corresponding to the time of maximum granuloma development (Figures 4 and 6).

**Figure 7:**
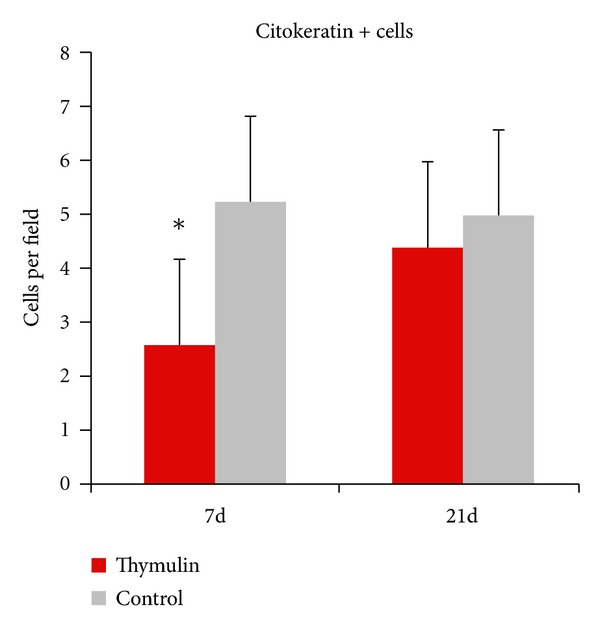
Cytokeratin positive phagocytes per field (mean) in the footpad, in function of time. Ten fields were counted per slide. *P = 0.01, Mann-Whitney, in relation to control.

**Figure 8:**
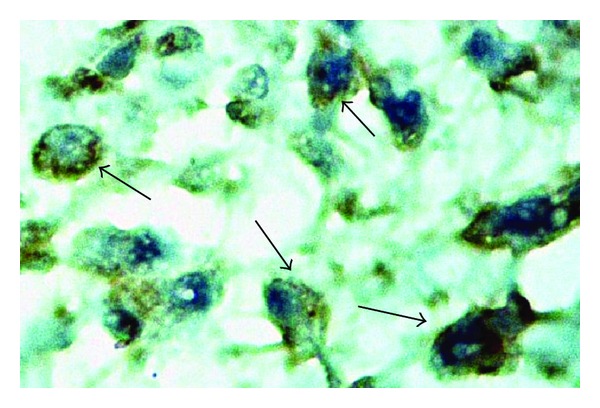
Photomicrography of cytokeratin positive phagocytes in the footpad. 7 day after BCG inoculation. Early epithelioid differentiation is seen in positive cells (arrows). Immunohistochemistry, magnitude 1 : 1000.

The histopathology of the granuloma analyzed in HE slides at the 7 day after inoculation presented less epithelioid cells and predominance of newly migrated phagocytes in thymulin 5CH treated mice in relation to control (Figure 6). The later evaluation of the lesion after the immunohistochemistry procedures using anticytokeratin antibody to identify mononuclear positive cells (epithelioid cells) confirmed, quantitatively, this effect. However, this difference was not prolonged to the 14 and 21 days (Figures 7 and 8, Table 2).

The number of CD34 positive cells per field in the footpad was also smaller in thymulin 5CH treated mice in comparison to the control group, but no difference between groups was seen regarding CD11b, CD3, and CD45R positive cells (Table 2).

**Figure 9:**
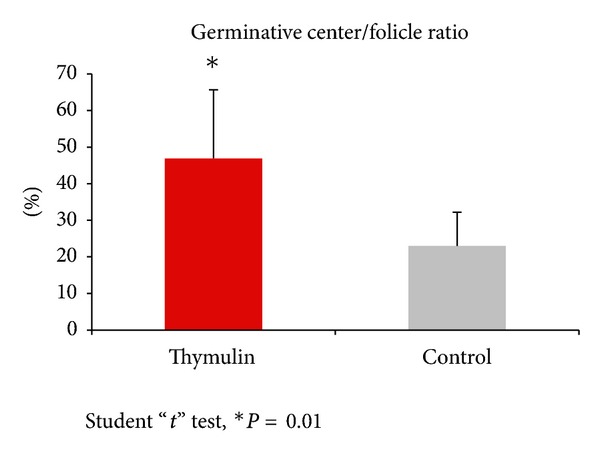
Histomorphometric analysis of germinal center/follicle diameter ratio (percentage) of the local lymph node. 21st day after BCG inoculation into the footpad. *P = 0.01, student “*t*” test.

The lymph node histomorphometry revealed increase in germinal center/follicle diameter ratio in thymulin 5CH treated mice compared to the control group (Figure 9).

**Figure 10:**
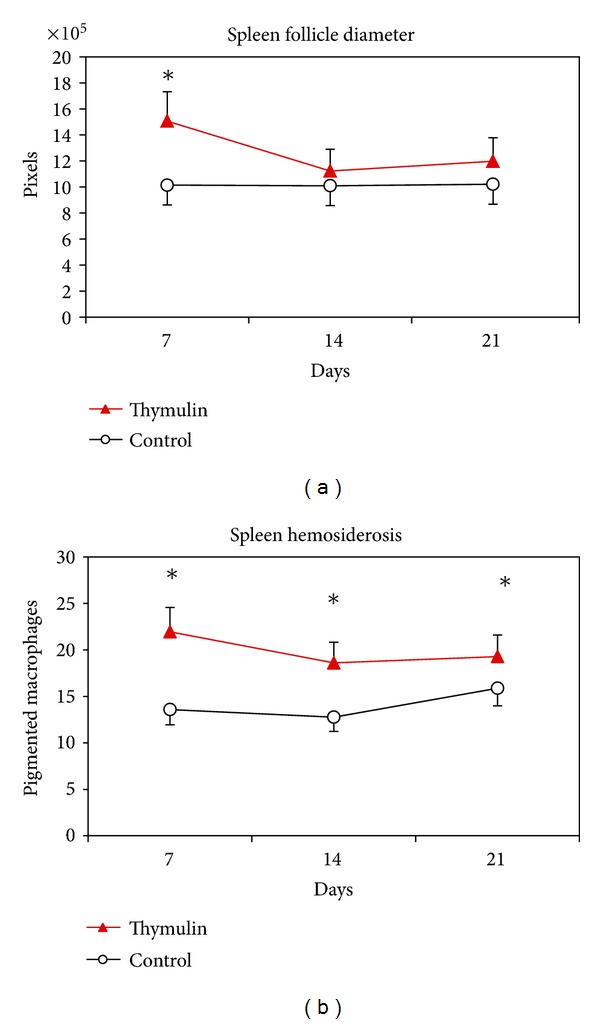
(a) Histomorphometric analysis of spleen, showing the mean of follicular area (pixels) and standard deviation, in function of time. (b) Pearls staining positive phagocytes for hemosiderin per field (mean, standard deviation) in the subcutaneous tissue of the footpad, in function of time. Ten fields were counted per slide. *P = 0.05, MANOVA.

The histomorphometrical analysis of the spleen showed increase in follicle diameter in thymulin 5CH treated mice at the 7 day after BCG inoculation, but not at the 14 and 21 days (Figure 10(a)). The Pearls staining method revealed increase in phagocyte activity of iron pigments in spleen in thymulin 5CH treated group during all experimental period (Figure 10(b)).

### 3.3. Flow Cytometry Analysis

#### 3.3.1. Peritoneal Washing

**Figure 11:**
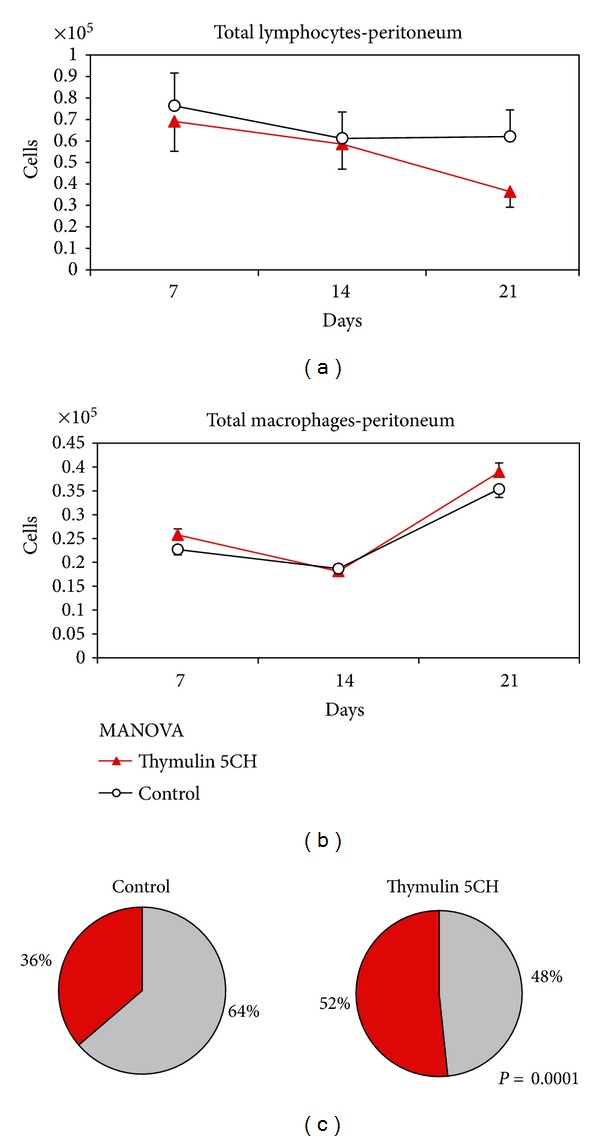
(a) Total peritoneal lymphocytes; (b) total peritoneal phagocytes, both quantified by flow cytometry in function of time. Data represented by mean and standard deviation, MANOVA; (c) proportion lymphocyte (grey)/phagocyte (red) at day 21st. Data represented by the mean of cells harvested from five mice per group. *P = 0.0001 in relation to control, *X* ^2^.

The analysis of peritoneal washing was split in two sets of data: (a) those from the lymphocyte gate and (b) those from phagocyte gate (Figure 3(a)). The total cell counting from each gate revealed an inversion of both populations in function of time, with increase of the population presenting phagocyte morphology (higher size and lumpiness) at the 21 day. This pattern was equal as in control as in thymulin 5CH treated group, but this last group showed more intense and significant inversion compared to the control (Figures 11(a), 11(b), and 11(c)).

**Figure 12:**
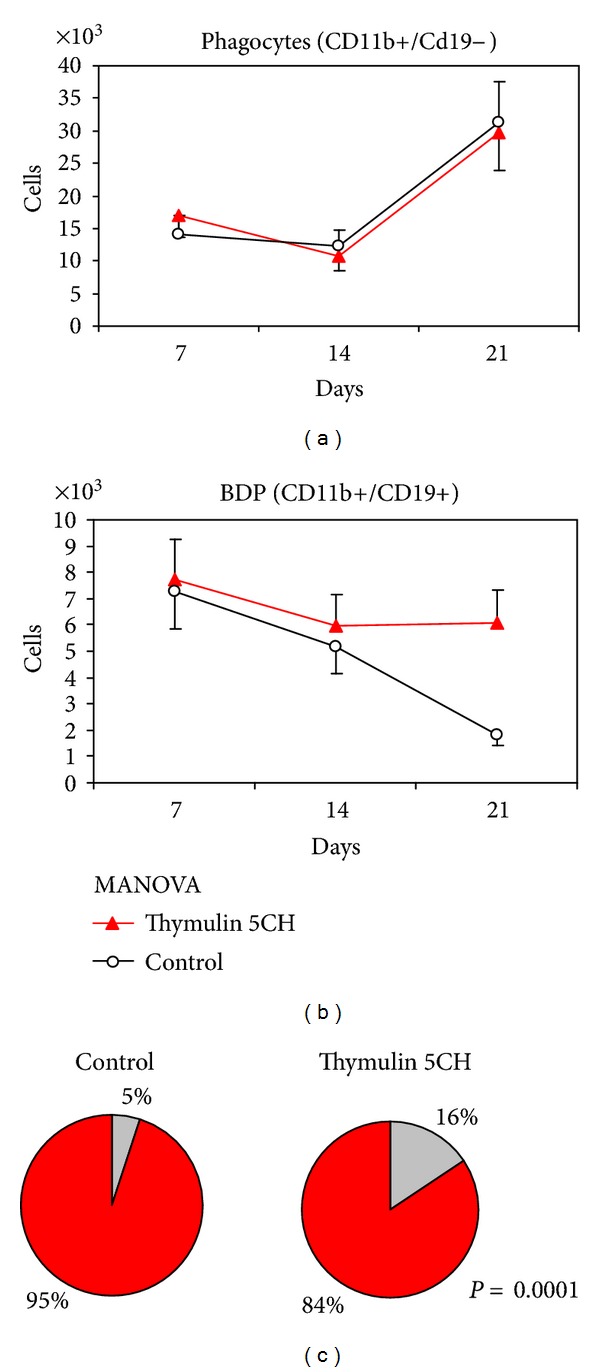
Phagocyte subsets in the peritoneum. (a) number of CD11b+/CD19− mature phagocytes; (b) number of CD11b+/CD19+ double marked phagocytes (BDP), both quantified by flow cytometry in function of time. Data represented by mean and standard deviation. MANOVA. (c) BDP CD11b+/CD19+(grey)/mature phagocyte CD11b+/CD19−(red) proportion at day 21. Data represented by the mean of cells harvested from five mice per group. *P = 0.0001 in relation to control, *X* ^2^.

**Figure 13:**
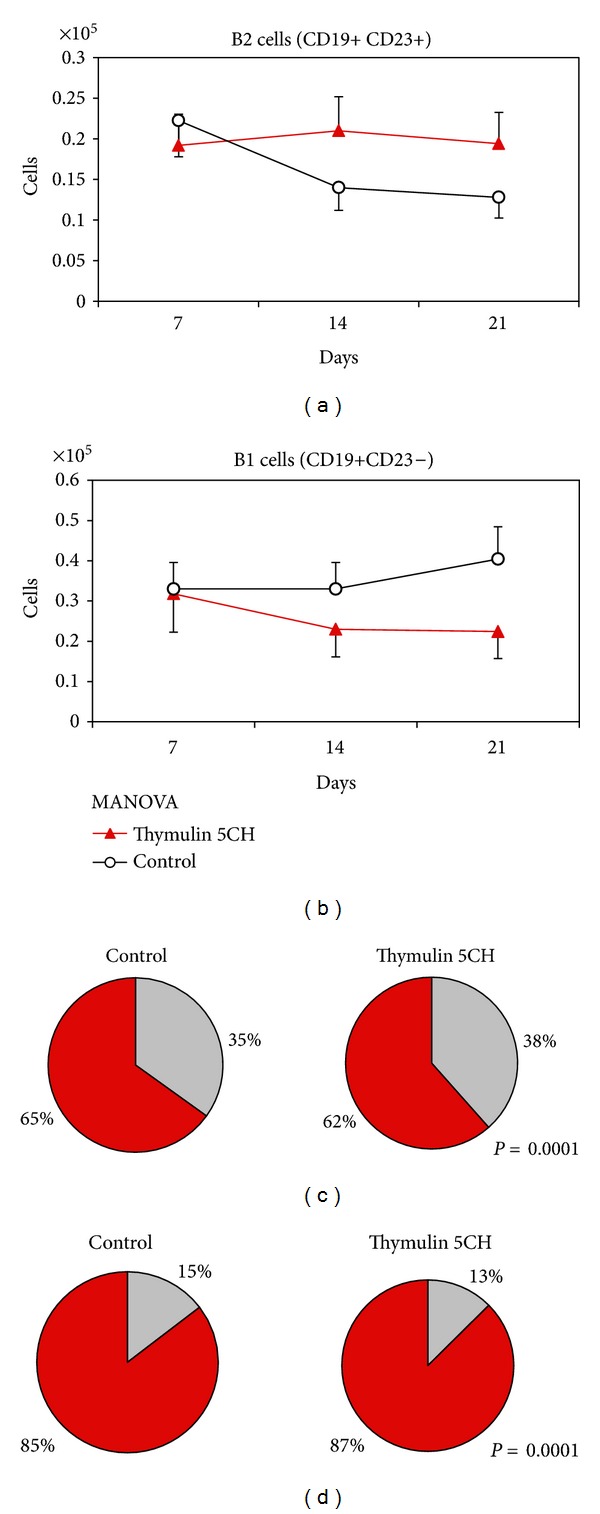
B-lymphocyte subsets in the peritoneum. (a) number of CD23+/CD19+ B2 cells; (b) number of CD23−/CD19+ B1 cells, both quantified by flow cytometry in function of time. Data represented by mean and standard deviation. MANOVA. (c) other lymphocytes (grey)/B1 (red) proportion; and (d) B1a (grey)/B1b (red) proportion at day 21. Data represented by the mean of cells harvested from five mice per group. *P = 0.0001 in relation to control, *X* ^2^.

The analysis of specific phagocyte gate shows a peak of mature phagocytes at 21 day for both groups (Figure 12(a)) and thymulin 5CH treated mice showed a steady percentage of B1-derived phagocytes (BDP) at this time, different to the control (Figure 12(b)). Thus, the proportion between BDP/mature phagocytes was highlighted in treated animals (Figure 12(c)). Instead, the lymphocyte gate showed the inverse behavior: the B1 population was reduced at day 21 in thymulin 5CH treated mice in contrast to the control. The B2 cells kept their population steady in function of time (Figures 13(a), 13(b), and 13(c)). Also, a mild reduction in B1a (CD19+/CD5+)/B1b (CD19+/CD5−) ratio was seen in thymulin 5CH treated animals after 21 days from BCG inoculation (Figure 13(d)).

This opposite behavior between B1 cells in the lymphocyte population and B1 cells in the phagocyte population of the peritoneal cavity indicates the B1 differentiation into mature phagocytes.

#### 3.3.2. Lymph Node Cell Suspension

Since the main effects of thymulin 5CH on peritoneal B1, B2, and phagocytes were got after 21 days from BCG inoculation into the footpad, the analysis of cell suspension harvested from popliteal local lymph node was focused on this time. Data were also split in two sets: (a) those from the lymphocyte gate and (b) those from phagocyte gate. From the lymphocyte gate, a supplementary data was obtained by counting T-cells subsets CD19− CD4+ or CD19− CD8+ (Figures 3(b) and 3(c)).

**Figure 14:**
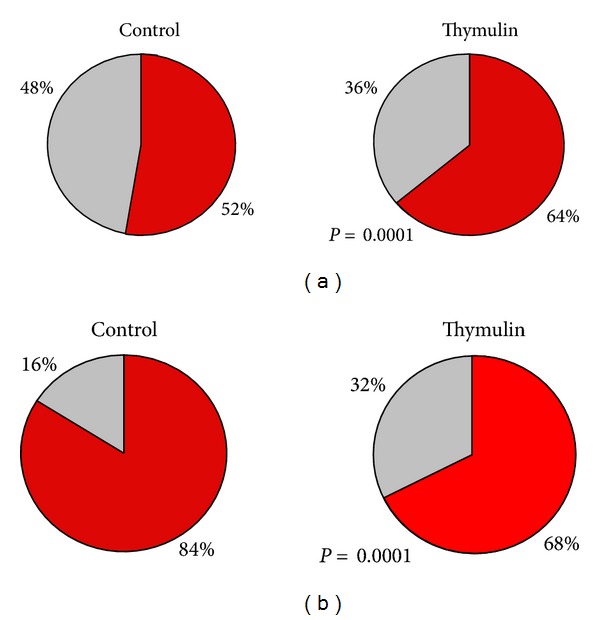
Lymphocytes subsets obtained from the lymphocyte gate after flow cytometry. Samples harvested at the 21 day after BCG inoculation. (a) Proportion between B1 CD11b+/CD19+ cells (red) and other lymphocytes (grey); (b) proportion between B2 CD11b−/CD19+ cells (red) and T CD11b−/CD19− cells (grey). Data represented by the average of both blocks of duplicate samples. *P = 0.0001 in relation to control, *X* ^2^.

**Figure 15:**
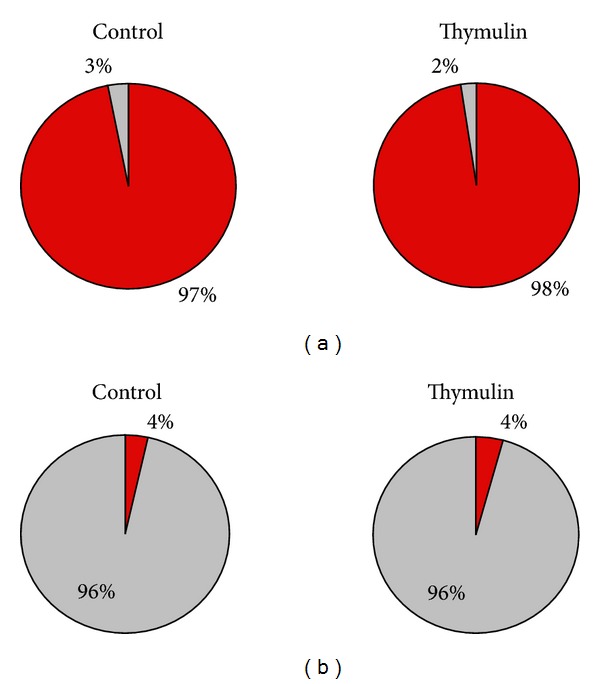
B1- and T-lymphocyte subsets obtained from the lymphocyte gate after flow cytometry. Samples harvested at the 21 day after BCG inoculation. (a) proportion between B1a CD11b+/CD19+/CD5+ cells (red) and B1b CD11b+/CD19+/CD5− cells (grey); (b) proportion betwen T CD4+ cells (red) and T CD8+ cells (grey). Data represented by the average of both blocks of duplicate samples, *X* ^2^.

The balance of cells subsets present in the lymphocyte gate has shown that thymulin 5CH treated mice presented more double positive B1 (CD11b+ CD19+) and double negative T (CD11b− CD19−) cells regarding to B2 (CD11b− CD19+) cells, comparing to the control (Figures 14(a) and 14(b)). No difference was seen in relation to B1a/B1b ratio or in relation to CD4+/CD8 ratio (Figures 15(a) and 15(b)). Traces of T reg CD25+ cells were seen in both groups, without statistical significance between groups (Thymulin 5CH = 1.75 ± 0.957; control = 1.5 ± 1.914 CD25+ cells in 10000 events).

**Figure 16:**
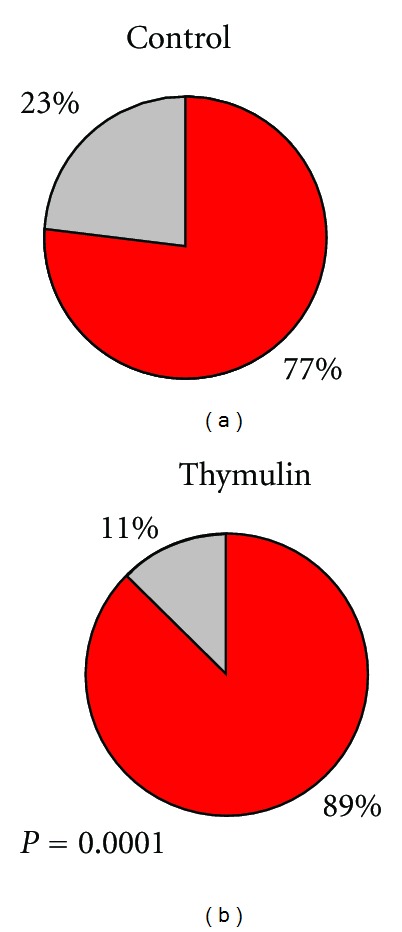
Cells obtained from the phagocyte gate after flow cytometry. Samples harvested at the 21 day after BCG inoculation. (a) Proportion between BDP CD11b+/CD19+ cells (red) and other phagocytes CD11b+/CD19− (grey). *P = 0.0001 in relation to control. Data represented by the average of both blocks of duplicate samples, *X* ^2^.

The analysis of phagocyte gate also showed an increase of double positive BDP (CD11b+ CD19+) cells in relation to other phagocytes (CD11b+ CD19−) in thymulin 5CH treated group (Figures 16(a) and 16(b)).

## 4. Discussion

Mice treated with thymulin 5CH presented a particular kinetic of cell migration toward the BCG-induced granuloma. In the first seven days, more young mononuclear cells and less epithelioid cells were identified in the inflammation focus, in comparison to the control, which was confirmed by immunohistochemistry. At the same time, higher phagocytic activity was observed in spleen macrophages too, whose pattern lasted up to the 21 day. This indicates that thymulin 5CH treatment is able to modulate the central and local innate immune response since its early phase. In spleen, probably the local macrophages were the responsible to this increase in phagocytic activity, but the hypothesis of B1-cells migration from the peritoneum to the spleen followed by phagocyte differentiation cannot be ignored [39, 40].

B-1 cells constitute a unique B-cell population with distinct ontogenic, phenotypic, and functional characteristics. These cells found preferentially in peritoneal and pleural cavities of adult mice are partially similar to B-2 cells because of the expression of CD19, IgM, and IgD but not CD23. B-1 cells have been described as a promiscuous cell lineage because of the co-expression of myeloid markers, such as CD11b. The expression of CD5 determines two subtypes of B-1 cells: B-1a (CD5+) e B-1b (CD5−) [41–44]. Different groups have already demonstrated that B-1 cells can differentiate into mononuclear phagocytes and to migrate to inflammatory foci *in vivo*, suggesting the involvement of these cells in essential immune mechanisms, as inflammatory responses [36, 45–47].

In the current study, after 21 days from BCG inoculation, the most intense innate immune response modifications were seen. Treated mice presented exuberant gross subcutaneous lesion and the phenotypic features of the present cells showed less CD34+ bone-marrow-derived cells per field, comparing to control, corroborating the hypothesis of phagocyte migration from alternative hematopoietic sites to the local infection, which decreased faster in treated than in control mice.

In the local lymph node of thymulin 5CH treated mice, higher germinal center/follicle diameter ratio could also be seen, suggesting lymphocyte collaboration in the local response regulation. In fact, at the flow cytometry analysis, an increase in the T-cells population was seen in relation to B cells. On the other hand, an increase of B1 cells could also be seen in relation to the other lymphocyte subtypes. In the phagocyte population, treated mice showed more BDP (CD19+/CD11b+ phagocytes) [34] among them, comparing to the control. Maybe in control group the main source of phagocytes had been the bone-marrow-derived monocytes or even the same B1 peritoneal cells in a slowly migration kinetic, in order to give time to migrated cells to lack its ability to express CD19, as have been described before [35, 45]. Considering that B-1 cells are the main source of B-cell-derived IL-10 [41], a putative modulation role of B1 cells in the lesion site over the other phagocytes in thymulin 5CH treated mice cannot be disregarded, probably by paracrine secretion of IL-10 [48, 49]; this hypothesis deserves further investigation.

At the same time, 21 days, in the peritoneum, the balance of resident cells showed more macrophages than lymphocytes in treated mice. Analyzing each population, it could be seen more B1-derived phagocytes and less B1 lymphocytes, suggesting phagocyte differentiation of B1 stem cells. It is corroborated by the increase in the percentage of B1b cells in relation to B1a cells. It is known that B1b cells can differentiate spontaneously into phagocyte *in vitro* [34] and *in vivo* [46], even though B1a can also do it when properly stimulated by inflammatory signals [50–52]. On the other hand, both B1a and B1b could also secrete antibodies (IgM, IgA), naturally (B1a) or signaling-induced (B1b) [44, 47].

Taking the data together, it seems that, thymulin 5CH triggers a sequence of coordinated systemic physiological effects. During the 21 days of observation, the trend of B1 peritoneal cells to differentiate into phagocyte and to migrate into local lymph node, near to the subcutaneous granuloma, is more evident in thymulin 5CH treated mice. Besides the natural site of B1 cells is the peritoneum, no B1 cell is seen in lymph nodes in basal conditions [53]. However, the characterization of a solid population of B1 cells in the local lymph node (Figure 3(b)) indicates their migration to this site in treated mice. The peritoneal B1-cells migration to remote inflammation sites, including BCG-induced granuloma cell population is well described in the literature, [38, 45, 54], as well as the higher phagocytic/antigen presentation activities in phagocytes derived from peritoneal B1 cells in mice [55].

The increase of local lymph node T lymphocytes also suggests some collaboration of these cells in this process and the final result is the improvement of infection remission, as seen in Figure 5. Indeed, T-cell collaboration to B1 cells was already described in other pathological conditions, such as tissue allograft rejection and experimental visceral leishmaniasis [56]. Regarding the B2 peritoneal cells, thymulin 5CH treated mice presented a trend to keep its levels constant, avoiding the decline observed in the control. Although the mechanism of B2 control by thymulin 5CH remains unknown, a natural hypothesis that emerges from our results is the possibility to have at least part of the peritoneal B1 cells differentiated into B2 cells, instead of phagocytes. It is known that this kind of differentiation can be done *in vivo*, according to the inflammatory signals context, for instance, in absence of galectin-3 or in the presence of signals mediated by Toll-like receptors [57, 58].

The modulation action of homeopathic thymulin preparations over lymphocytes is known since the eighties [15, 59], although very few articles are found in the literature about and little is known about which methods of thymulin preparation were used and how was the zinc concentration. About the use of nonhomeopathic very low doses of serum thymic factor, classical studies performed in radiation-induced immune deficiency show that low doses are more effective than higher ones [60, 61], however, in these studies, doses varied from 3 to 500 micrograms. The effects observed in the present followed a quite lower dose than those described before: thymulin 5CH (4 × 10^−13^ M or 0.4 ng in the water bottle) was offered into the drinking water reaching a dose equivalent to 4 pg/mouse, which means, 10^9^ times smaller than that described by [22] (10 mg/mouse). Also, differently to the formers, they were prepared according to the official homeopathic method, as described in specific pharmacopeias. How these homeopathic ultra high dilutions act upon biological systems is still unknown, but some recent studies suggest the participation of nanostructures in homeopathic preparations beyond Avogadro's number [62, 63]. Further studies about the determination of a putative ideal dose/homeopathic potency of serum thymic factor in mice challenged with BCG or other infectious agents can still be done, using potency-effect correlations, even including those above 10^−23^ M [4].

Additionally, the present results still open a new and original point of view about thymulin 5CH effect: the ability of homeopathic very high diluted zinc-free serum thymic factor to increase phagocyte differentiation from mouse peritoneal B1 cells.

Recently, an experimental study performed by our group in a commercial broiler chicken breeding revealed the impact of the immune modulation effect of thymulin 5CH on the lymphoid organs histology and on the productivity index (based on weight gain), showing an interesting usefulness of homeopathic preparations as a zoo technical tool [13]. Besides serum thymic factor, high dilutions effects on inflammation by other endogenous and exogenous immune modulating substances were also described, such as bursin [64], glucocorticoid [11, 65], and plant extracts [66, 67]. All these substances, when in high diluted homeopathic preparation, may represent potential tools for these technical purposes.

The global analysis of the results corroborate the previously described immune modulating effect of high diluted thymulin 5CH [13] and adds important new information about the granuloma cell influx control and the phagocyte efficiency, observed by the BCG infection remission. Although B1 cells have not been described in humans, the present results also invite us to consider the putative benefits of homeopathic high diluted serum thymic factor when associated to BCG human vaccination or to traditional treatment of natural *Mycobacterium* sp infection.

## Figures and Tables

**Table 1:** Markers used in the immunohistochemistry.

Marker	Cell	Supplier	Molecular target	Clone	Origin species/target	Dilution
Anti-CD34	Marrow stem cells	Serotec	CD34	Mec14.7	Rat-mouse	0.5%
Anti-CD3	T cells	Serotec	CD3	CD3-12	Rat-mouse	1 : 80
Anti-CD11b	Migrating phagocytes	Serotec	CD11b	M1/70.15	Rat-mouse	10%
Anti-CD45R	B Lymphocytes (specifically)	Serotec	Surface protein (LCA)	RA3-6B2	Rat-mouse	0.5%
Anti-cytokeratin	Epithelioid cells	Serotec	Poly-cytokeratin	Polyclonal	Rabbit-mouse	10 *μ*g/mL

**Table 2:** Cell counting per field using different cell markers. Ten fields per slide of footpad connective tissue were counted in blind.

Marker	Control	Thymulin 5CH
Anti-CD34 (day 21)	1.787 ± 0.750	1.368 ± 0.541*
Anti-CD3 (day 21)	1.600 ± 1.510	2.400 ± 0.540
Anti-CD45R (day 21)	0.800 ± 0.830	0.400 ± 0.540
Anti-CD11b (day 21)	1.765 ± 0.780	1.419 ± 0.587
Anti-cytokeratin		
Day 7	5.275 ± 2.172	2.600 ± 1.195*
Day 21	5.020 ± 2.759	4.42 ± 1.486

Data represent mean ± standard deviation. *P = 0.01, Mann-Whitney in relation to control.
